# Depression and the Link with Cardiovascular Disease

**DOI:** 10.3389/fpsyt.2016.00033

**Published:** 2016-03-21

**Authors:** Arup K. Dhar, David A. Barton

**Affiliations:** ^1^Human Neurotransmitters Laboratory, Baker IDI Heart and Diabetes Institute, Melbourne, VIC, Australia; ^2^Alfred Psychiatry, Alfred Health, Melbourne, VIC, Australia; ^3^Faculty of Medicine, Nursing and Health Sciences, Monash University, Melbourne, VIC, Australia

**Keywords:** depression, coronary heart disease, cardiac risk, cardiovascular disease, major depressive disorder

## Abstract

This review provides an outline of the association between major depressive disorder (MDD) and coronary heart disease (CHD). Much is known about the two individual clinical conditions; however, it is not until recently, biological mechanisms have been uncovered that link both MDD and CHD. The activation of stress pathways have been implicated as a neurochemical mechanism that links MDD and CHD. Depression is known to be associated with poorer outcomes of CHD. Psychological factors, such as major depression and stress, are now known as risk factors for developing CHD, which is as important and is independent of classic risk factors, such as hypertension, diabetes mellitus, and cigarette smoking. Both conditions have great socioeconomic importance given that depression and CHD are likely to be two of the three leading causes of global burden of disease. Better understanding of the common causal pathways will help us delineate more appropriate treatments.

The known major links between CHD and MDD are outlined in this review. The search terms “depression, major depressive disorder, coronary heart disease, cardiac risk, cardiovascular disease,” were entered into an electronic database, “PubMed.” Articles published between 1950 and 2015, and written in English were chosen. Abstracts were then hand screened for relevance and were selected on the basis of addressing mechanisms associating MDD and CHD.

## Depression and Coronary Heart Disease Prevalence

Major depression is a debilitating condition that presents with a number of cognitive and biological symptoms, including a pervasively lowered mood, anhedonia, negative cognitions, anergia, and appetite disturbance, and at its worst can manifest itself with suicidal thoughts and acts and psychotic features (1). The lifetime prevalence rates of major depression are in the order of 17%. It is known that major depression is more prevalent in people who have suffered a major cardiac event, with up to 40% of patients meeting the criteria for major depressive disorder (MDD) (2). The large European Action on Secondary Prevention through Intervention to Reduce Events (EUROASPIRE) study showed potentially even higher rates with up to 35% of men and up to 65% of women measured as having depression on the hospital anxiety and depression scale (3). The enhancing recovery in coronary heart disease (ENRICHD) trial looked at patients who had recently suffered a myocardial infarction (MI), and depression was diagnosed in 74% of them (4). If we look at figures from the community through to those who are hospitalized, we see rates of depression of 10% in general practice clinics (5, 6), which then increases to up to 30% in those with coronary heart disease (CHD) in outpatient clinics (5–8), and up to a staggering 50% in those who are an inpatient for coronary artery bypass surgery (6, 9).

The leading cause of mortality in the developed world is coronary artery disease (CAD). When cardiac disease and major depression present together, the prognosis for both worsen (10–12). Reviews have continually shown that major depression is associated with poorer quality of life (13) and increased morbidity (14–16) and mortality (7–9, 17, 18). If MDD is present at baseline in a CHD patient, then it is an independent risk factor for poorer cardiovascular outcomes including MI (16).

A meta-analysis of 11 studies showed that MDD conferred an overall relative risk of 1.64 for developing CHD (19). The severity of the depression is proportional to the risk of developing CAD (20). Regarding mortality, MDD confers a relative risk of 1.8 in those suffering comorbid CHD (21). For those who have suffered a MI, the presence of MDD is a bad prognostic factor and carries a five times increased risk of cardiac mortality within 6 months (22).

## Mechanisms of Major Depression and Increased Cardiac Risk

**Figure 1:**
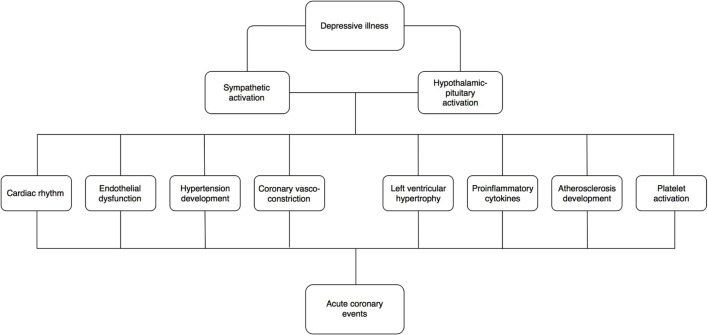
**Possible mechanisms whereby depression confers elevated cardiac risk**. This is likely to be multifactorial including sympathetic activation, hypothalamic–pituitary activation, endothelial dysfunction, platelet activation, proinflammatory cytokines, and atherosclerosis development along with cardiac vascular and rhythm abnormalities.

It has long been accepted that there are numerous behavioral and lifestyle factors at play that confers increased CHD risk in those suffering depression. Psychological stress experienced by people suffering from MDD can also cause deregulation in the sympathetic nervous system and hypothalamic–pituitary–­adrenal (HPA) axis (23, 24) (see Figure 1). This in turn has a number of deleterious downstream effects, including the development of hypertension, left ventricular hypertrophy (25), coronary vasoconstriction, endothelial dysfunction (26–28), platelet activation, and the production of pro inflammatory cytokines (29, 30) (see Table 1). The potential consequence of this is an elevated risk in ventricular arrhythmias (31) and MI (32).

**Table 1:** **Summary of main findings in mechanisms of MDD and cardiac risk**.

Reference	Objective	Subjects	Methods	Results
Stapelberg et al. (33)	To identify a model of causal mechanisms that link major depressive disorder to cardiovascular heart disease	–	A comprehensive literature review	Mechanisms linking MDD and CHD that are often discussed in the literature are genetic, behavioral, and immunological. As well as coagulation, omega-3 PUFA deficiency and vascular endothelial dysfunction. However, rather than discussing them separately, these mechanisms should be viewed as interdependent networks
Esler et al. (23)	To examine the sympathetic nervous system function in depression	11 depressed patients	Tritiated norepinephrine was used to observe the rate of norepinephrine spill over. Removal of norepinephrine from plasma was performed to assess the neuronal uptake of norepinephrine	Norepinephrine spill over was elevated in 5 of the 11 patients with depressive illness. Rapid removal phase of norepinephrine from plasma corresponded in patients with depression, suggesting increased neuronal uptake
		8 patients with other psychiatric diagnosis		
		17 healthy subjects		
Carney et al. (34)	To examine whether depressed patients with coronary artery disease (CAD) have lower heart rate variability compared with non-depressed CAD patients	19 depressed CAD patients	Patients underwent 24-h Holter monitoring, with SD of normal-to-normal intervals were used as the primary index of heart rate variability	Heart rate variability was significantly lower in non-depressed CAD patients
		19 non-depressed CAD patients		
Spieker et al. (28)	To identify the mechanisms behind endothelial functioning in response to mental stress	23 healthy subjects	Nitroglyercin and flow-mediated (FMD) induced vasodilation were examined pre- and post-stress. FMD was also examined during stress	Endothelium-dependent vasodilation decreased by half in comparison to endothelium-independent vasodilation to nitroglyercin which remained unaffected. Intra-arterial infusion of norepinephrine for a similar duration of mental stress did not hinder FMD
Schlaich et al. (35)	To determine possible mechanisms leading to sympathetic augmentation in hypertension	22 hypertensive patients	Microneurography and radio tracer dilution were used to measure regional sympathetic activity	Patients with hypertension demonstrated reduced neuronal norepinephrine reuptake, which in turn was associated with sympathetic activation in hypertension
		11 normotensive patients		
Dinan (36)	To review the biological markers of depression, specifically neuroendocrine disturbances and the hypothalamic–pituitary–adrenal axis (HPA)	–	A comprehensive literature review	Numerous studies have demonstrated that MDD results from activation from the HPA. Other biological markers may be secondary to high glucocorticoid levels
Wong et al. (37)	To examine centrally directed norepinephrine and corticotropin-releasing hormone (CRH) secretion in medication-free patients with melancholic depression	10 depressed patients	Norepinephrine and CRH were measured in lumbar cerebrospinal fluid every 30 h in addition to plasma adrenocorticotropic hormone and cortisol secretion	Depressed patients had significantly higher levels of norepinephrine and plasma cortisol yet normal levels of CRH. There was a negative correlation between plasma cortisol and CRH in healthy patients, while there was no relationship demonstrated in the depressed group
		14 healthy subjects		
Barton et al. (38)	To assess the significance of the sympathetic nervous system in the promotion of cardiac risk in patients with MDD	39 depressed patients	Whole-body cardiac sympathetic activity was measured using noradrenaline isotope dilution methods and sympathetic nerve recording techniques	A bimodal distribution for whole body and heart sympathetic activity was demonstrated in patients with MDD. This subset of MDD patients had significantly high sympathetic activity
		76 healthy subjects		
Ghiadoni et al. (27)	To explore whether mental stress promotes atherogenesis *via* the impairment of endothelium-dependent vascular homeostasis in preclinical subjects	8 non-insulin-dependent diabetic patients	Response to sublingual glyceryl trinitrate and brachial artery flow-mediated dilation (FMD) were measured before and after a mental stress test. It was measured again in the healthy group, without a stress stimuli on a separate occasion	In healthy subjects, mental stress had no effect on the response to sublingual glyceryl trinitrate; however, FMD significantly reduced after. Without the stress stimuli, FMD was unchanged. While the diabetic group had lower FMD in comparison to controls, there were no changes following the stimuli for both FMD and sublingual glyceryl trinitrate responses
		10 healthy subjects		
Brown et al. (6)	To review the associations between MDD and CHD and address the clinical implications for treatment	–	A comprehensive literature review	While there is no evidence that treatment of depression will reduce this effect, it is still important to improve quality of life for those with depression through the benefits of psychological and pharmaceutical intervention
Poole et al. (39)	To review the relationship between depression and adverse outcomes from acute coronary syndrome (ACS) and coronary artery bypass graft (CABG) surgery patients	–	A comprehensive literature review	Inflammation is a frequent causal process involved in the development of depressive symptoms and for adverse cardiac outcomes

## Behavioural and Lifestyle Factors

It is well established that there are number of behavioral and lifestyle factors, which are present in MDD patients that can increase the chance of developing CHD (40). These include increased rates of smoking, alcohol intake, physical inactivity, and obesity (33). As seen with other medical conditions, major depression predicts poorer adherence responses in CHD patient to medications (41), lifestyle (42–44), and rehabilitation programs (45). In fact, non-completion rates in cardiac rehabilitation have been shown to be in the order of 44% compared to 29% in the non-depressed group (46). Therefore, we are likely to see the depressed CHD patient being less motivated and adherent to cardiac rehabilitation programs. MDD also makes it less likely to engage in lifestyle modification after a major cardiac event to reduce the classic risk factors of CHD. We see this with regards to cigarette smoking, where MDD patients are less likely to give up smoking and there consumption is also heavier (47).

## Disturbance in Autonomic Nervous System

It has long been known that “stress” and heart disease are linked (10, 48). Stressful events, such as terrorist attacks (49), natural disasters (50), and even high stakes knockout soccer matches, have been positively associated with an increase in acute cardiovascular events (51). Mental stress is one of the many cognitive symptoms that people with major depression suffer from. Laboratory mental stress tests have been shown to activate sympathetic nervous outflow in the non-cardiac patient setting (52). The cardiac sympathetic nerves are preferentially activated by such mental stress (11). In animal models and the clinical setting (31, 53), the significance of the activation of cardiac sympathetic cardiac fibers has demonstrated disturbances in heart rhythm, leading to increased risk of ventricular arrhythmias, decreased blood flow (54), left ventricular hypertrophy (25), and MI and sudden death (28). It has been shown that in depressed CHD patients, there is an elevated resting heart rate, and this also may be due to sympathetic hyperactivity in this group of patients (34).

Thus, it is postulated that the lowered removal of noradrenalin from the cardiac sympathetic synapse enhances the sympathetic stimulation and as a consequence confers CHD risk (38). In fact, it has been shown that there is a bimodal distribution of noradrenalin spill over in patients with MDD, with approximately one-third of patients exhibiting remarkably high levels (38). Interestingly, it has been shown that there is an elevated serotonin turnover in MDD patients who have not yet been treated with antidepressants, which is associated with the short allele of the serotonin transporter (55). This short allele has been also associated with increased urinary noradrenalin levels and therefore sympathetic over activity (56).

Hypertension is an established risk factor for the development of CHD. Up to 90% of patients with heart failure suffer from hypertension (28). Essential hypertension is often triggered and maintained by mental stress. The processes here include reduced uptake of noradrenalin (35) and activation of brain noradrenergic pathways and noradrenalin release from the heart. It has been demonstrated that there is indeed elevated noradrenalin levels in depressed patient’s plasma and cerebrospinal fluid (57). We have seen other psychogenic causes of hypertension with the well-known phenomenon of white coat hypertension, which persists after numerous visits (58), where you would traditionally expect some form of desensitization to occur and blood pressures to normalize.

With regards to the parasympathetic system, a further possible mechanism linking MDD and CHD includes heart rate variability as a marker of vagal activity (59). Decreased heart variability (60–62) is associated with post-infarct mortality (63–66). Heart rate variability is modulated by the cardiac vagus and has been described in MDD patients (10).

## Disturbance of the Hypothalamic–Pituitary–Adrenal Axis

Major depressive disorder has been implicated in the stress-induced activation of the HPA axis (36). Elevated levels of cortisol have repeatedly been found in MDD patients (37, 67, 68). This hypercortisolemia causes an increased risk of a metabolic syndrome type state, which includes glucose intolerance, hyperlipidemia, and increased visceral fat mass (69–71). This metabolic syndrome not only confers a higher risk of cardiovascular disease (72) and diabetes (73) but has also been shown to drive sympathetic activation (74).

## Platelet, Inflammatory and Autoimmune Mechanisms

Stress and anxiety, both common symptoms of major depression, can contribute to atherosclerosis (38). Increased platelet activation and endothelial dysfunction have been implicated as a potential pathophysiological pathway linking MDD and CHD (75, 76).

Prolonged mental stress, which is commonly experienced in those suffering from major depression, has been shown to induce prolonged endothelial dysfunction (27, 28). This endothelial dysfunction has been shown to be one of the early signs of future cardiovascular deterioration (77). In fact, oxidative stress itself has been described in numerous psychiatric illnesses (78).

Antiplatelet medication, such as aspirin, has long been a mainstay of preventative treatment of CHD (79). As stated, depression has been implicated with increased platelet reactivity (80), which will increase the relative risk of thrombus formation and arterial occlusion (10). MDD is associated with raised platelet serotonin levels, which promotes clotting (81). Further evidence of platelet activation can be shown by elevated levels of ­beta-thromboglobulin, which are raised in depressed CHD patients (80). It is this prothrombotic state, which leads to thrombus formation and consequently myocardial ischemia (17).

Inflammatory pathways have been proposed as one of the causal pathways responsible for MDD causing increased CHD risk (39), *via* atherosclerosis (82). Depressed patients have been found to have elevated levels of inflammatory markers, such as C-reactive protein (CRP), and proinflammatory cytokines, such as interleukin 1, 2, 6, and tumor necrosis factor (83–87). A number of these inflammatory markers have also been consistently associated with poor cardiovascular outcomes (88), and CRP can predict MI (89).

## Implications for Treatment

Once major depression is present in those who have had a major cardiac event, we can expect them to still be suffering from depressive symptoms 4 months post discharge from hospital (90). It is important to recognize depression in CHD, yet the majority of cases are not diagnosed or managed accordingly (91). In a consensus statement from the National Heart Foundation of Australia, they recommended that routine screening for depression is carried out for all patients suffering from CHD and first presentation and at follow-up (92). A perceived barrier to screening for depression by clinicians is lack of time (93). A quick and simple way to screen for depression in this setting would be to use a validated rating scale, such as the Cardiac Depression Scale (94).

The onset of a major depression has been shown to negatively moderate treatment outcomes for CHD patients (95). A recent Cochrane review showed that antidepressant medication was superior to placebo in the treatment of major depression in those who are also physically unwell.

Of the various antidepressants available to treat MDD, it is not known which is the best to lower the risk of CVD events in those suffering MDD (96). Common side effects of antidepressants must be taken into consideration when prescribing in those depressed CHD patients. This includes dizziness, appetite changes, sleep disturbance, gastrointestinal side effects, and sexual side effects.

Selective serotonin reuptake inhibitors (SSRIs) are the first-line pharmacotherapy for the treatment of major depression. This is generally because of their tolerability and safety profile (97, 98) and are deemed to be safe in the cardiac setting (6, 99). The SSRIs have been shown to have a meaningful effect on major depression (100). The Sertraline Antidepressant Heart Attack Randomized Trial (SADHART) demonstrated that the antidepressant sertraline improved depression in those who had suffered acute coronary syndrome (ACS) (101). The safety and efficacy of sertraline in the CHD group was evidenced in this study (101). The ENRICHD trial showed that antidepressant treatment improved CHD prognosis (102). In the review by (103), it was shown that antidepressant medication can positively alter physiological pathways linking MDD and CHD. We have stated that sympathetic over activity is important in causality of CHD, and reductions in sympathetic activity have been shown following SSRI treatment (104). However, SSRIs are not without their flaws, and it has been noted that some induce weight gain and metabolic abnormalities (105–109).

There are various classes of antidepressants, such as the aforementioned selective SSRIs, tricyclic antidepressants (TCAs), and monoamine oxidase inhibitors (MAOIs). They are equally as efficacious (110), but differ significantly in their receptor and this side effect profiles. The TCAs and MAOIs are generally avoided in the treatment of major depression in those who have comorbid cardiac conditions, because of their toxic cardiac side effects (111). TCAs have been associated with elevated relative risk of 1.24 for CHD (112) and increased mortality rates in CHD (113). TCAs are also are also highly toxic in overdose (114), which is of concern to the prescribing clinician especially when treating those with a history of suicidal behavior. TCAs can cause QTc prolongation (115) and decrease heart rate variability (64) secondary to their anticholinergic effects.

With regards to psychological therapies for depression, such as cognitive behavioral therapy (CBT), they seem to be efficacious for the depression without being beneficial for CHD morbidity or mortality (116). The UK National Institute of Clinical Excellence’s (NICE) guideline on depression in adults with chronic physical health problems advises that psychological treatment (such as CBT) be the first line of treatment for mild to moderate depression (110). However, this may not hold true for CHD patients and certainly is not the case for those suffering a more moderate to severe form of major depression, where biological treatment, such as antidepressants, are favored.

Physical exercise has long thought to be beneficial for one’s mental state, and in the past, it was common to see exercise prescribed by their doctors, to aid with the treatment of major depression. A Cochrane review has indicated that moderate aerobic exercise in the order of 30 min, five times a week is beneficial for relieving MDD symptoms. The magnitude of its efficacy should not be dismissed as Cochrane showed it had similar efficacy to CBT (117), and it of course will be beneficial to one’s cardiovascular health. In those who exercise, it can be seen that depression, quality of life, and global functioning scores improve, thus decreasing the intensity and longevity of the episode of MDD (118). In the cardiac rehabilitation, setting exercise programs has not only shown to be beneficial for depression scores but also improves body percentage fat, triglyceride, and cholesterol levels (119).

In relation to platelet function, we have already discussed that platelet hyperactivity leads to a prothrombotic state, which in turn leads to increased risk of CHD. The SADHART platelet substudy showed a decreased platelet activation in those patients who had suffered an MI, were depressed, and had it treated with a SSRI, namely, sertraline (101). Paroxetine has also been shown to decrease platelet hyperactivity (120, 121). SSRIs have been shown to decrease sympathetic hyperactivity and, as a consequence, potentially reduce cardiac morbidity and mortality (38).

## Conclusion

The underlying mechanism linking MDD and CHD are complex and multifactorial. As discussed, they involve the sympathetic nervous system, platelet hyperactivity, inflammation, and HPA dysregulation among others. Given that a definitive mortality study is unlikely to be done due to the complexities and costs involved, we are therefore guided by current evidence to maximize efficacy and potential harm when choosing our treatments for MDD who have comorbid CHD. We should be considering MDD as a common and modifiable risk factor for CHD, just like we do for smoking, hypertension, hyperlipidemia, and the like. The toxic combination of MDD and CHD leads to poorer health outcomes for both conditions and escalating health-care costs. The implications for research is that further understanding of the pathophysiological mechanisms underpinning MDD and CHD is required so as to inform us of better treatments. It would be interesting to see not only studies looking at underlying biological mechanisms but also clinical ones. There needs to be an effort to try and minimize the barriers to screening and treating of MDD in those suffering from CHD. Given the bidirectionality of the conditions, it would also be useful to elucidate whether treatments for CHD can potentially decrease the intensity of symptom burden of MDD. Future clinical practice may include initiating cardioprotective medications alongside antidepressants when MDD is diagnosed, thus alleviating the morbidity, mortality, and cost burdens of MDD and CHD.

## Author Contributions

AD and DB made equal contribution to the preparation, revision and production of this review.

## Conflict of Interest Statement

The Human Neurotransmitters Laboratory is currently receiving research funding from National Health and Medical Research Council of Australia and from Otsuka Pharmaceuticals. It has previously received research funding from Servier Pharmaceuticals. Dr. AD has received travel honoraria from Servier and Otsuka pharmaceuticals. Prof. DB has received honoraria for presentations from Eli Lilly, Servier, Pfizer, Solway Pharmaceuticals, Wyeth Pharmaceuticals, Lundbeck, and AstraZeneca. He has received research funding from Servier, Pfizer, Eli Lilly, Otsuka, and Lundbeck.
